# Top economics universities and research institutions in Vietnam: evidence from the SSHPA dataset

**DOI:** 10.1016/j.heliyon.2021.e06273

**Published:** 2021-02-17

**Authors:** Quan-Hoang Vuong, Anh-Tuan Bui, Manh-Toan Ho, Thanh-Hang Pham, Thi-Hanh Vu, Hung-Hiep Pham, Anh-Duc Hoang, Manh-Tung Ho, Viet-Phuong La

**Affiliations:** a Centre Emile Bernheim, Université Libre de Bruxelles, 1050 Brussels, Belgium; b Centre for Interdisciplinary Social Research, Phenikaa University, Hanoi 100803, Viet Nam; c Faculty of Business Administration, Foreign Trade University, Hanoi 100000, Viet Nam; d A.I. for Social Data Lab, Vuong & Associates, Hanoi 100000, Viet Nam; e School of Business, RMIT Vietnam University, Hanoi 100000, Viet Nam; f School of Economics and International Business, Foreign Trade University, Hanoi 100000, Viet Nam; g Center for Research and Practice in Education, Phu Xuan University, Hue 530000, Viet Nam; h EdLab Asia Educational Research and Development Centre, Hanoi 100000, Viet Nam

**Keywords:** Scientific collaboration, Scientific publishing, Economics discipline, Vietnam, Bayesian analysis

## Abstract

Economic research is vital for creating more suitable policies to facilitate economic growth. Employing a combination of descriptive and Bayesian analyses, this paper investigates the research landscape of the economics discipline in Vietnam, in particular, the leading affiliations in the field and how these institutions compare to each other in terms of productivity, the number of lead authors, new authors and publications' journal impact factor. We also examine the differences in the authors' productivity based on their age and gender. The dataset extracted from the SSHPA database includes 1,444 articles. The findings show that among top producers of economic research in Vietnam, seven are universities, leaving only one representative of research institutes. These top producers account for 52% of research output among 178 institutes recorded in the database. We also find a correlation between a researcher's affiliation, sex, and scientific productivity in Vietnam's economic discipline. Overall, publications by male researchers outnumber those by female ones in most of the top affiliations. The findings also indicate that 40–44 is the age group with the highest scientific productivity. Researchers' collaboration, which is observed through co-authorship, is on the rise in all of the top eight economic research affiliations. However, the quality of current Vietnam's scientific works in the discipline is questionable. Therefore, it is suggested that in order to sustain scientific productivity, economic researchers might need to balance the quantity and quality of their contributions.

## 1 Introduction

“*The curious task of economics is to demonstrate to men how little they really know about what they imagine they can design.*”

-*Friedrich August von Hayek*

Economic development is crucial to enhance social living standards, reduce the poverty rate, and increase the position of a country on the world map, especially for emerging countries. Vietnam, as an emerging economy, has witnessed strong growth in the economic sector. In the last ten years, as of 2018, more than 45 million Vietnamese are out of poverty, leading to a sharp decrease of the poverty rate from more than 70% to below 6% (USD 3.2/day according to purchasing power parity) (World Bank, 2019). Accordingly, the country's GDP per capita increases by 2.5 times, reaching over USD 2,500 in 2018. The development of the national economy is forecasted to remain robust.

Along with economic development, economic research is vital for undertaking more suitable economic policies so as to boost a stable and non-inflationary growth and help cope with various risks under a dynamic and rapid integration of an economy (Fullani, 2007). Therefore, parallel with the country's economic advancement is the significant development of economic researchers in the Vietnamese social sciences community. Recent findings from Vuong et al., 2020a, Vuong et al., 2020b, Vuong et al., 2020c, Vuong et al., 2020d indicated that economics has been the leading discipline among various areas in Vietnamese Social Sciences for the 2008–2018 period. In particular, within the 2011–2017 period, around 384 Social Sciences and Humanities (SSH) projects were funded by the Vietnam National Foundation for Science and Technology Development (NAFOSTED). Amongst these, around 24% of the projects belonged to the Economics discipline (95/384) (Nafosted, 2018).

To further develop economic researches to support decision-making for policymakers as well as practitioners, it is important to understand the characteristics of those studies and their authors, including their affiliations, age groups, sex, authorship role, or collaboration pattern. However, few studies focus on investigating such characteristics and their influence on Vietnamese economic researchers' productivity. Therefore, to better understand the research landscape of economics discipline in Vietnam, first, we would like to investigate the leading players in this area, including both universities and research institutes, as these are the main contributors to the development of this research field. Then, we want to examine how these top affiliations compare to each other in a number of aspects, including the number of lead and new authors as well as their publication's journal impact factor. Finally, we want to indicate whether there is a relationship between economists' scientific productivity and their sex or age within these top research producers. To answer these questions, a Bayesian analysis is employed on a dataset of 178 affiliations, and 1,444 articles count in three sub-categories, namely Business, Economics, and Management, during the period from 2008 to 2019. It should be noted here that the real number of articles may be different due to overlapped works within these sub-categories.

## 2 Literature review

### 2.1 Affiliations and scientific productivity

To date, not many studies have investigated the association between affiliation and scientific productivity. In a study by Allison and Long (1990), it was stated that productive scientists tend to work in prestigious university departments, and the results indicate that the effect of department affiliation on productivity is of higher importance to productivity than the reverse impact. Hayati and Ebrahimy (2009) stated that in terms of quantity, universities outnumber other research institutions, while there is no difference when it comes to quality.

In the context of Vietnam's social sciences, using a dataset of 657 Vietnamese social scientists, Vuong et al. (2019) found that authors working at universities have much higher scientific output than those affiliated with research institutions. In addition, the authors also point out that universities in Vietnam are more focused on teaching rather than engaging with research activities. In 2018, there were 454 higher education institutions in Vietnam, including 95 private universities and colleges, with the role of delivering education to about 2.2 million students (MOET, 2019). Even though the number of institutions is quite big, the research function of those institutions might not be fully exploited. Moreover, the government's investment in higher education is relatively low, and higher education is still struggling between being controlled by the government and being fully autonomous (Salmi and Pham, 2019). Therefore, the difference between researchers at universities and institutions in Vietnam is striking and needs further investigation.

### 2.2 Age and scientific productivity

Previous studies show inconsistent findings in the relationship between age groups and scientific productivity. A study from Costas et al. (2010) on a total of 1,064 Spanish National Research Council scientists in Spain, indicated that the productivity decreases in older scientists, especially the low-class researchers. A lack of resources or motivation is believed to be the explanatory factor for this declining trend in scientific performance. However, the authors suggest that the collaboration between scientists from different age groups can reduce the obsolescence and generation effects due to changes in cultural, social, and technical environments (Kyvik and Olsen, 2008). Given the ongoing development of society and modern technologies, older scientists might be able to catch up with new changes through such collaborations.

On the contrary, González-Brambila and Veloso (2007) used a unique data set of 14,328 researchers to explore the contributing factors of research output and impact. Even though the authors confirm a quadratic relationship between age and the number of published papers, they suggest that this factor does not have a substantial influence on research output and impact. They propose a new publishing peak of the Mexican researchers, which is approximately 53 years old. However, they claim that the increased publishing peak is not a major issue in terms of the count of publications. In Italia, Abramo et al. (2015) suggested seniority has a positive relationship with productivity, especially in medicine or biology. A more recent study suggested that Italian academia has become more progressive, despite seniority still having a big influence (Marini, 2017). Based on a Scopus dataset with publication profiles of 410 Vietnamese researchers between 2008 and 2017, a previous study claims that the most crucial group of authors contributing to the Vietnamese research community within the last few years is the seniority group. They are around 40–50 years old and often play the first-author role in the researches (Vuong et al., 2017).

These inconsistent results show that there needs to be more investigation into the correlation between age and scientific productivity within various research contexts. As a Confucian culture (Vuong et al., 2018a, 2020a), seniority certainly has a big influence on Vietnamese society. However, scientific development often borns out of radical young minds. Institutional policies, as well as governmental support for scientists, are issued at specific times. Certain age groups might benefit more from them. Thus, age and scientific productivity is a unique relationship that should be examined.

### 2.3 Sex and scientific productivity

Women's contribution to science is crucial to social development; however, sex difference has been affecting the quantity and quality of scientific performance for a long time (Sotudeh and Khoshian, 2014). Despite some improvements, women are still suffering from the sex gap and biases in the science world. Previous researches find that there are sex discrepancies in research funding, productivity, and impact. An analysis of Québec university professors in Canada by Larivière et al. (2011), indicated women above 38 years old receive less funding for research than men on average. This leads to generally less productivity in terms of publications and less scientific impact for female researchers. According to the authors, possible explanations include limited networks, motherhood, division of labor, and the scientific community's hierarchy.

On the other hand, using the scientometric method with a comparative approach, the study by Sotudeh and Khoshian (2014) indicated female researchers’ positive performance in the Nano Science & Technology series. The information of these female authors is extracted from eighteen journals in the field listed in the Journal Citation Report. The sample size included 13,491 researchers. According to their findings, although female Nano-researchers are scarce in number, they equally perform in terms of scientific productions and impacts, which imply sex equality in the Nano field particularly.

Also, the female-to-male ratio in research productivity has been found to increase from about 60% in the late 1960s to around 80% in the late 1980s and early 1990s (Xie and Shauman, 1998). Moreover, in the context of the United States, Xie and Shauman (1998) also observed that most of the sex differences in research productivity can be attributed to personal characteristics, structural positions, and marital status. Those results suggest that sex differences in research productivity stem from sex differences in structural locations.

Apart from the publication rate, citation rate is also one of the key points to evaluating sex equality in scientific performance. Based on a dataset of 8,500 Norwegian researchers and more than 37,000 publications, Aksnes et al. (2011) concluded that women's publications are less cited than are those of men. However, sex inequality in citation rates can be attributed to differences in scientific productivity.

Last but not least, according to the EuropeanCommission (2013) report, although the youngest generations of female academics have been receiving more support, the sex gap is still disproportionately high compared with the increase in the proportion of women students. This thus casts doubt on the hypothesis that women will automatically ‘catch up’ to their male counterparts in scientific productivity. The issue also requires further investigation to shed light on it.

In the Asian context, several studies have attempted to investigate the impacts of sex on scientific productivity and found conflicting results. For instance, the work by (Tao et al., 2017), with a sample of 30,078 participants in China, discussed women's underrepresentation in both science and engineering in the country. On the other hand, in Russia, the proportion of female scientists reaches parity compared to their male counterparts (Huang et al., 2020).

### 2.4 Authorship, collaboration, and scientific productivity

Using publication data from the Norwegian national database – FRIDA, a dynamic authority record, covering 19,000 controlled scientific and scholarly publication channels and the four major research universities in Norway as the scope of the research, Piro et al. (2013) found the importance of collaborative research in the performance of scientific research. Another study by Cainelli et al. (2015) also claimed that increasing extensive collaboration is a common behavior in the scientific community. Lissoni, Mairesse, Montobbio, and Pezzoni (2011) found that the size and international nature of collaborative projects and co-authors’ past productivity have significant impacts on current productivity, while age and gender, and past productivity are also influential determinants of both productivity and probability of promotion.

From a recent study by Ho et al. (2017), among Vietnamese scientists who have published in indexed international journals, there have been signs of low sustainability, such as the lack of information distribution in the co-authorship network or a high level of reliance on a few highly connected members in the networks. This study also aims to investigate this co-authorship pattern among Vietnam's top institutes in the economics discipline.

### 2.5 Journal impact factors and scientific productivity

Despite the controversy, the Journal Impact Factor (JIF) is still one of the most widely used indicators of quality (Van Leeuwen and Wouters, 2017). Devising by Eugene Garfield, the IF provides a proxy to quantify the scientific ranking and journal prestige of a scientific journal (Grech and Rizk, 2018). The IF is, in fact, a functional approximation of the mean citation rate per citable item. Despite the popular application of IF, it has also been criticized heavily. Scientific journals naturally flaunt their high IFs to attract a higher number of publications from which they can pick and choose and thereby further increase their IF (Grech and Rizk, 2018; Shanta et al., 2013). The IF can be cheated by purposely exploiting publication bias or citation bias (Evangelou et al., 2012). Moreover, the costs of publishing in a high IF journal is large (Jain, 2016). The costs include the large time investments researchers have to prepare a good manuscript and go through multiple rounds of reviews and revisions.

In Vietnam, JIF has been gaining popularity as an indicator to evaluate the quality of publications. The introduction of NAFOSTED brings about the initial motivation to Vietnamese scholars to publish on ISI/Scopus indexing databases in general. It contributes to the race to publish in international journals, preferably with a high impact factor. Various universities also use JIF as the criteria for the bonus; for example, the University of Economics Ho Chi Minh City (UOE HCM) gives a cash bonus of up to USD 8,600 for a research article published in a journal that has JIF above 2 (Vu, 2017). Therefore, this paper wants to look at the comparison among top universities and research institutes in Vietnam's economic discipline in terms of their publications' JIF.

Overall, a substantial body of literature focusing on analyzing researchers’ scientific productivity has been shown in many previous studies. However, there are still inconsistent results that need further investigation, especially from emerging economies like Vietnam. Hence, this paper will shed light on scientific productivity in one of the leading social science fields in the country, namely economics.

### 2.6 Research questions

To achieve the research aims, we will answer a list of specific questions, as listed in Table 1.

**Table 1:** Research questions.

Characteristics of the data	Questions	Method used
Affiliation	1. What are the top universities and research institutes in Vietnam's economic discipline? 2. How are the top universities and research institutes' productivity levels compared to each other?	Descriptive data analysis Descriptive data analysis & Bayesian data analysis
Age	3. Is there any difference in the productivity of the authors based on their age? 4. What is the difference between the age of male and female authors?	Descriptive data analysis Bayesian data analysis
Sex	5. Is there any difference in the productivity of the authors based on their sex? 6. Is there any relationship between authors' scientific productivity and their sex?	Descriptive data analysis Descriptive data analysis
Lead, New Authors & Co-authorship	7. How are top universities and research institutes in economics compared in terms of the number of lead authors? 8. How are top universities and research institutes in economics compared in terms of the number of new authors? 9. How are top universities and research institutes in economics compared in terms of co-authorship?	Descriptive data analysis Descriptive data analysis Descriptive data analysis
JIF	10. How are top universities and research institutes in economics compared to their publication's journal impact factor?	Descriptive data analysis

## 3 Materials and methods

### 3.1 Materials

A comprehensive dataset of Vietnamese researchers' scientific productivity in the economic fields from 2008–2019 was extracted from the Social Sciences and Humanities Peer Awards (SSHPA) database. SSHPA's function is to record the scientific productivity of Vietnamese Social Sciences and Humanities (SSH) researchers as a semi-automatic database (Vuong et al., 2018b). The used dataset is deposited on Open Science Framework (La et al., 2020), containing the authors' observations from 178 affiliations and articles in the economic fields. The papers are categorized into three sub-fields: Business with 361 papers, Economics with 930 papers, and Management with 153 papers. However, the number of articles in these sub-fields might be overlapped. The list of affiliations is drawn by article, only taken from level 1 affiliations; the output is calculated plus sub-affiliations for level 1 affiliations. Demographic and academic characteristics such as age, gender, new authors, leading authors, co-authorship, and impact factors are also considered. Raw data were then entered into a Microsoft Excel spreadsheet, cleaned, and saved in .csv form.

### 3.2 Methods

This study employed a combination of a descriptive data analysis and a Bayesian analysis to answer the research questions. Accordingly, for the latter approach, a hierarchical regression model of the number of published articles according to sex and affiliations is developed by using R statistical software and the bayesvl package (v0.9.5). The bayesvl package is available on The Comprehensive R Archive Network (La and Vuong, 2019). It allows facilitating new knowledge precisely without traditional meta-analyses and yields more principled conclusions from each new study (Kay et al., 2016). It is proposed that the Bayesian analysis can be used as an alternative approach for the conventional frequentist approach in analyzing social data based on its advantages of treating all unknown quantities probabilistically and incorporating prior knowledge or belief of scientists into the model (Vuong et al., 2020a). It can also help mitigate some shortcomings of the frequentist statistics, for example, the controversial issue related to how to interpret the “*p*-value” (Vuong et al., 2020b). This technique, which visually demonstrates results and distributions of coefficients, is relatively suitable for this study. When the model does not show sensitivity to adjustment of the prior, its credibility is proven (Scutari and Denis, 2014). Hence, the Bayesian statistics approach is used in this study to examine the relationship between sex, affiliation, and scientific productivity of the researchers in the dataset.

The variables ‘scientific productivity’ was analyzed as the main dependent variable in this study. The analysis would also include the following independent variables:

- “Article”: Number of publications
- “Age”: Age of the authors at the published time
- “Sex”: The biological sex of the respondents, with two categories “male” and “female.”
- “Affil”: The affiliation of authors
- “SexAffil”: For multi-layer partitioning, we need to combine the Affiliation variable with the Sex (biological gender) variable to create a new variable, which is SexAffil = Sex + “_” + Affil. The variable consists of 8 affiliations related to sex, ranging from 1_1–8 and 2_1–8, as shown in Table 2 below. The code to create this variable can be found in the Supplementary.

**Table 2:** Coded variables.

	SexAffil	Sex	Affiliation
1	1_1	Male	[1] (FTU) Foreign Trade University
2	1_2	Male	[2] (HCMOpenUni) Ho Chi Minh City Open University
3	1_3	Male	[3] (NEU) National Economics University Hanoi
4	1_4	Male	[4] (TMU) Thuongmai University
5	1_5	Male	[5] (UOEH) University of Economics Ho Chi Minh City
6	1_6	Male	[6] (VASS) Vietnam Academy of Social Sciences
7	1_7	Male	[7] (VNUH) Vietnam National University Hanoi
8	1_8	Male	[8] (VNUHCM) Vietnam National University Ho Chi Minh City
9	2_1	Female	[1] (FTU) Foreign Trade University
10	2_2	Female	[2] (HCMOpenUni) Ho Chi Minh City Open University
11	2_3	Female	[3] (NEU) National Economics University Hanoi
12	2_4	Female	[4] (TMU) Thuongmai University
13	2_5	Female	[5] (UOEH) University of Economics Ho Chi Minh City
14	2_6	Female	[6] (VASS) Vietnam Academy of Social Sciences
15	2_7	Female	[7] (VNUH) Vietnam National University Hanoi
16	2_8	Female	[8] (VNUHCM) Vietnam National University Ho Chi Minh City
- “Sexid”: the identity of the authors. Variable [1] is male and [2] is female

The coded variables in the dataset are described in detail in Table 2 below.

## 4 Results

To make it clear and easy to follow, we present the results as answers to the lists of questions presented in Table 1.

### 4.1 Descriptive analysis

***RQ1: What are the top universities and research institutes in Vietnam's economic discipline?***

From the dataset, there are eight universities and research institutions in the top list of the economic field. Among the total number of 178 universities and institutions, this group contributes to approximately 52% of total research output. The detailed list of these top affiliations with the number of articles published and the number of authors is presented in Table 3.

***RQ2: How are the top universities and research institutes' productivity levels compared to each other?***

**Table 3:** Top 8 Vietnam institutes have the highest productivity in economics research.

#	Institutes	Articles	Authors
1	National Economics University Hanoi	167	108
2	University of Economics Ho Chi Minh City	124	55
3	Vietnam National University Hanoi	121	81
4	Ho Chi Minh City Open University	72	47
5	Vietnam National University Ho Chi Minh City	60	37
6	Foreign Trade University	57	47
7	Thuongmai University	40	34
8	Vietnam Academy of Social Sciences	37	40

Interestingly, within these top 8 affiliations, there is only one research institution, namely the Vietnam Academy of Social Sciences, which contributed merely 2% to the total research output of the economics discipline. Compared to the first ranked position, the National Economics University, with a 13% contribution, this shows a substantial gap in productivity. It is also notable that the first three universities accounted for nearly one-third of all published articles by Vietnamese economists, with much higher productivity than the rest of the list.

**Figure 1:**
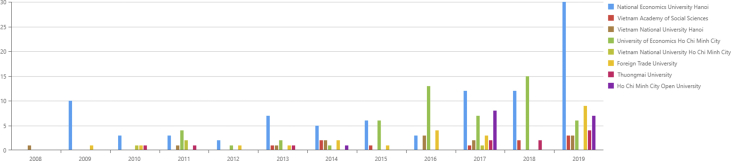
Number of publication of Top 8 affiliations 2008–2019.

When these top affiliations are compared over the years, the results are presented in Figure 1.

Results indicate that NEU's scientific productivity among the top eight institutes shows the most significant improvement, especially in 2017 and 2019. In 2017, we also witnessed an increase in the number of universities publishing articles. However, scientific productivity is a lack of stability and consistency. For instance, some affiliations have publications only in 2014, 2017, and 2019, such as Vietnam National University Hanoi (VNUH) or Thuongmai University (TMU). Such institutes have only several years of contributing scientific products within the 12 years; however, they are still leading the Vietnamese economics research enterprise.

**Figure 2:**
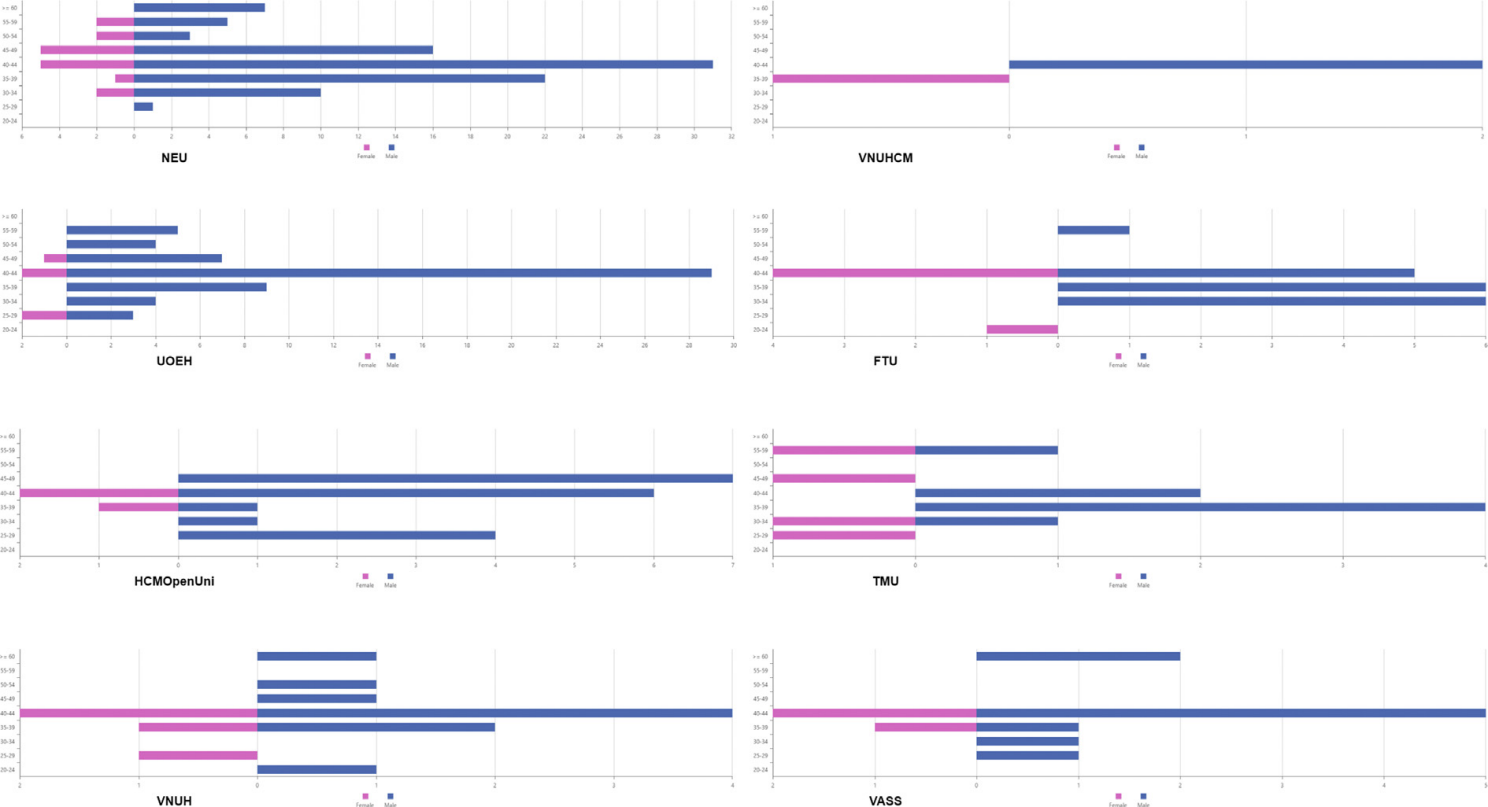
Age group and sex of the authors.

Research questions from 3 to 6 investigate the impacts of age and sex on scientific productivity among Vietnamese economists within the top affiliations. The results are presented in Figure 2 as follows.

Figure 2 shows the difference in the total number of articles from each university according to age groups and sex. The result shows that 40–44 is the age group with the highest scientific productivity in the vast number of affiliations in the 2008–2019 period. However, the younger groups (25–29, 30–39 age group) also contribute a great number of articles within the period and become the highest group of scientific contributors. For instance, Thuongmai University (TMU) has the highest number of articles in the 35–39 age group, or Foreign Trade University (FTU) has its highest one in the 30s group.

Figure 2 above also indicates the sex differences in the scientific productivity of the top eight affiliations in Vietnam. The number of articles from male authors outweighs the number of articles from females in most of the universities, especially in the University of Economics Ho Chi Minh City (UOEH), National Economics University Hanoi (NEU) – two in eight affiliations with the highest number of published articles. In those eight affiliations, the oldest age group of female authors is 55–59 years old, while male authors are above 60. Moreover, the age range of female authors is shorter than that of male authors.

***RQ7: How are top universities and research institutes in economics compared in terms of the number of lead authors?***

**Figure 3:**
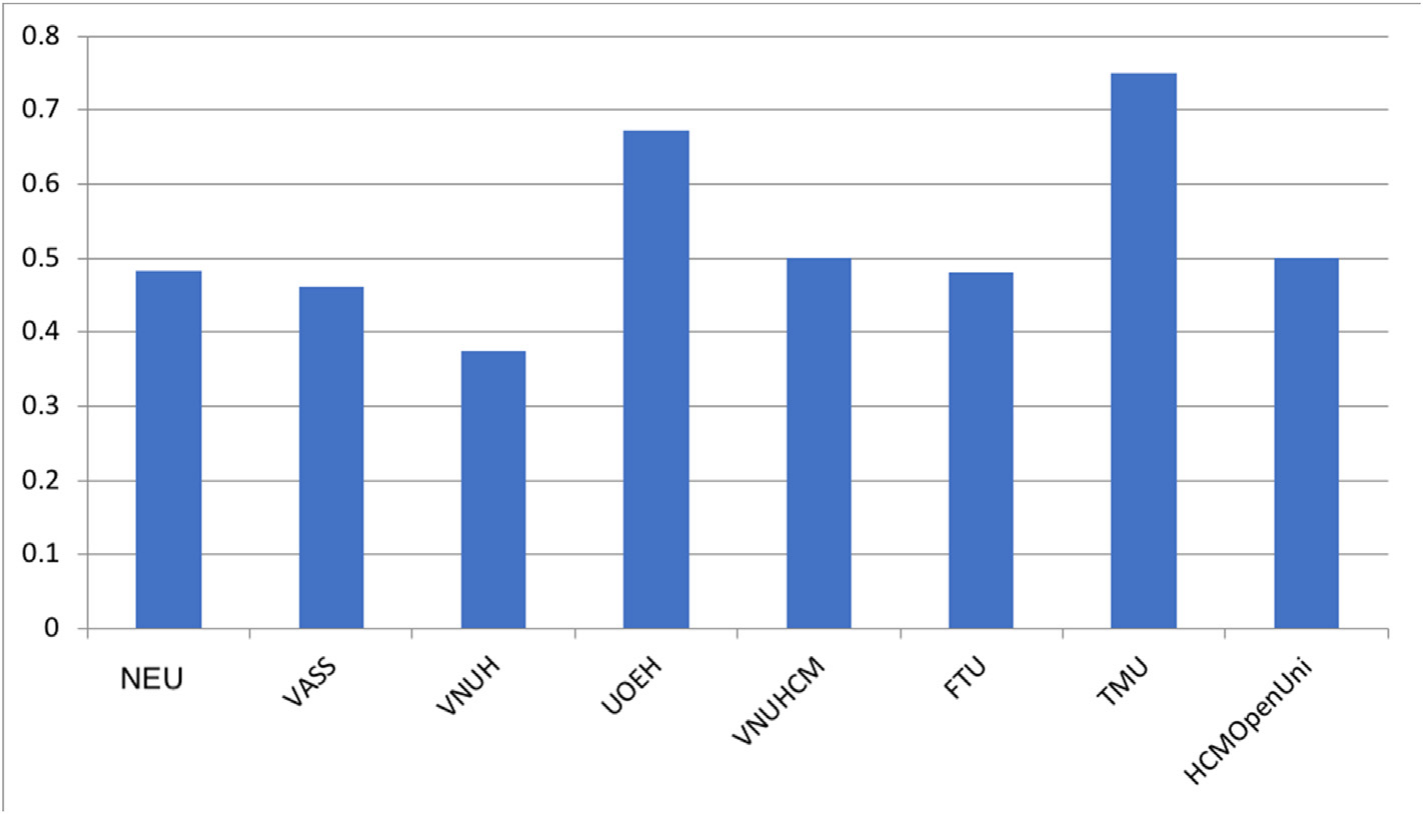
The ratio of lead authors per affiliation.

Figure 3 presents the number of lead authors in the top 8 affiliations, which can be categories into three groups: TMU and UOEH with the highest number of lead authors (more than 0.7); VNUH with the lowest number of lead authors (below 0.4); and the others have a similar rate of lead authors (roughly 0.5). Since 2017, the number of lead authors in NEU increases per year, significantly rockets to 21 authors in 2017 with 17 published articles.

***RQ8: How are top universities and research institutes in economics compared in terms of the number of new authors?***

**Figure 4:**
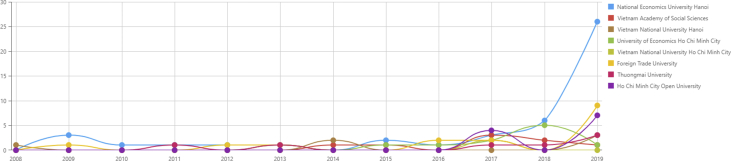
New authors per year.

In Figure 4, within the 2008–2016 period, there is only a small increase in the number of new authors per year. However, in 2017, there has been an explicit increase of new authors in many affiliations, such as Ho Chi Minh City Open University (HCMOpenUni). Thereafter, except for NEU and UOEH, other affiliations witness a decrease in the number of new authors in 2018. There is an upward growth of new authors in most affiliations in 2019, especially NEU, with more than 25 authors, contrasting with the decrease in UOEH.

***RQ9: How are top universities and research institutes in economics compared in terms of co-authorship?***

**Figure 5:**
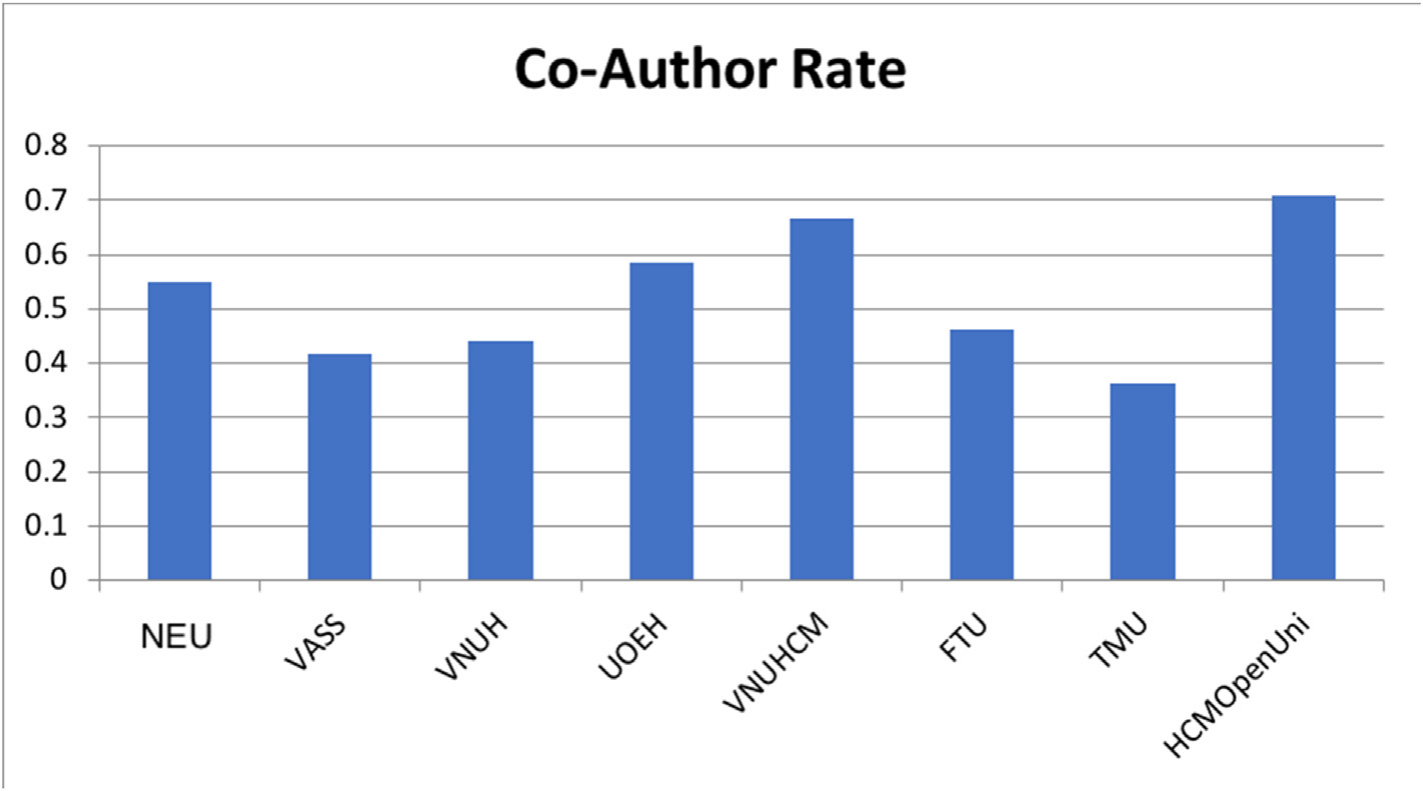
The average rate of co-authors per affiliation.

Findings from Figure 5 indicate that the closer the co-author rate is to 1, the less the number of co-authorships with outside sources of the affiliation. Accordingly, we have HCMOpenUni as the affiliation with the lowest of co-authorships (rate = 0.7) and TMU with the highest number of co-authorships (rate = 0.36). The results show NEU, UOEH, VNUHCM, and HCMOpenUni have high co-author rate, and VASS, VNUH, FTU, and TMU have low co-author rate (roughly 0.4).

***RQ10: How are top universities and research institutes in economics compared in terms of their publication's journal impact factor?***

**Figure 6:**
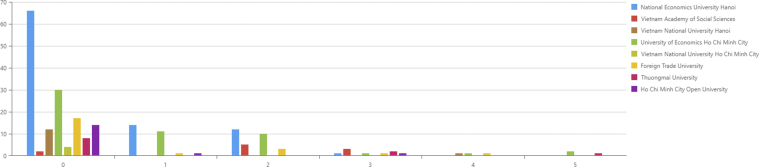
Articles per affiliation according to the Impact Factor.

Figure 6 shows that a significant number of articles from these eight affiliations have no JIF and only belong to Scopus or Emerging Sources Citation Index (ESCI). With JIF = 0, which means journals that do not have JIF, the highest number of articles comes from NEU, and the lowest number of articles comes from VASS. The number of articles above JIF = 3 is relatively low. Only UOEH and Thuongmai University have articles with JIF = 5 among the top list of affiliations.

### 4.2 Bayesian analysis

The formula of the Article model is as below:

*article ~ α[sexaffil*_*varint*_*] + age*

**Figure 7:**
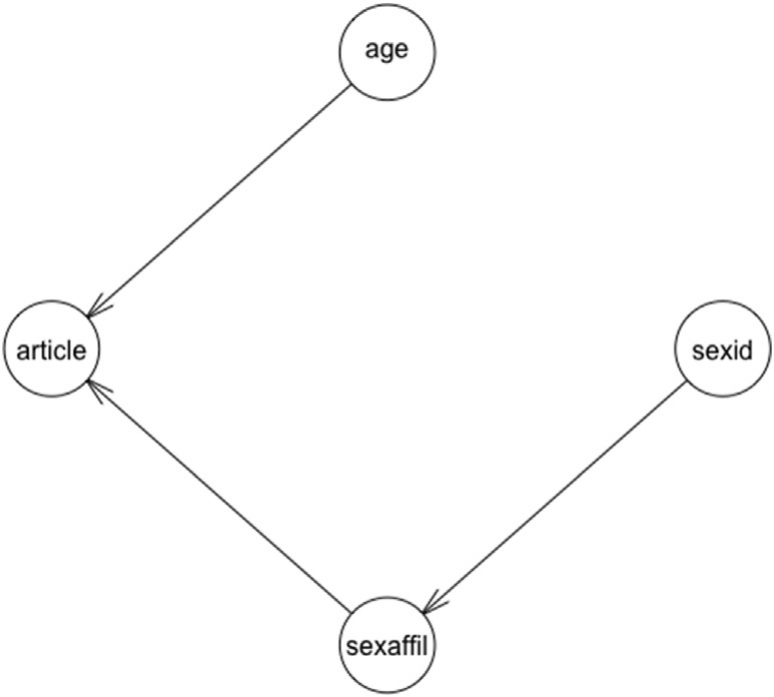
Visualization of the bayevl regression Article model.

The code that was used to construct the model Article is available in the Supplementary. Figure 7 presents the network of the Article model for the probabilistic dependency among the variables.

The STAN code will be automatically generated by bayesvl. Using the following command, we commenced the MCMC simulation process:

model < - bvl_modelFit(model, data1, warmup = 2000, iter = 5000, chains = 4, cores = 4)

The result of the model analysis is presented in Table 4:

**Table 4:** Summary of the bayevl regression Article model.

Variable	Mean	Se_mean	Standard Deviation	N_eff	Rhat
b_age_article	-0.03	0.00	0.02	5643	1
a_sexaffil[1]	3.02	0.01	0.85	9827	1
a_sexaffil[2]	3.26	0.01	0.87	9397	1
a_sexaffil[3]	4.32	0.02	0.95	3997	1
a_sexaffil[4]	2.90	0.01	0.91	4496	1
a_sexaffil[5]	3.86	0.01	0.86	5349	1
a_sexaffil[6]	2.95	0.01	0.91	10005	1
a_sexaffil[7]	3.02	0.02	0.92	2565	1
a_sexaffil[8]	3.11	0.03	1.03	1399	1
a_sexaffil[9]	2.71	0.03	0.97	987	1
a_sexaffil[10]	2.61	0.03	1.01	1144	1
a_sexaffil[11]	2.91	0.01	0.90	5965	1
a_sexaffil[12]	2.51	0.01	0.96	4580	1
a_sexaffil[13]	2.45	0.02	0.94	1512	1
a_sexaffil[14]	2.59	0.01	0.97	7129	1
a_sexaffil[15]	2.46	0.01	0.93	6218	1
a_sexaffil[16]	2.60	0.03	1.08	6218	1
sigma_sexaffi	0.69	0.01	0.27	1680	1
a_sexid[1]	3.28	0.01	0.82	5820	1
a_sexid[2]	2.63	0.02	0.85	1983	1
a0_sexid	2.71	0.07	2.97	1779	1
sigma_sexid	3.28	0.07	3.75	2716	1

**Figure 8:**
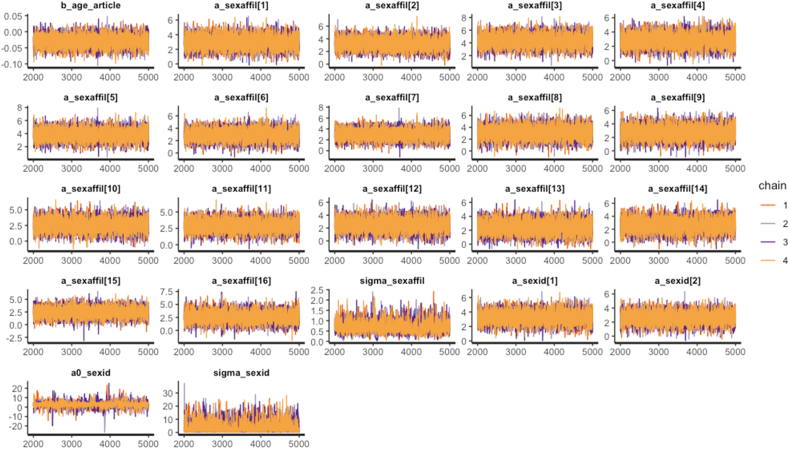
The MCMC chains for the Bayesian model of Article.

The summary of the model shows that Rhat is around 1 (more than 1.1 means problem), and *n*_eff is above 2000 (more than 1000 means good sign). Moreover, in Figure 8, we also can see that the convergence of our model is good.

The results of Bayesian analysis help to clarify answers to the research questions as follows:

***RQ2: How are the top universities and research institutes' productivity levels compared to each other?***

**Figure 9:**
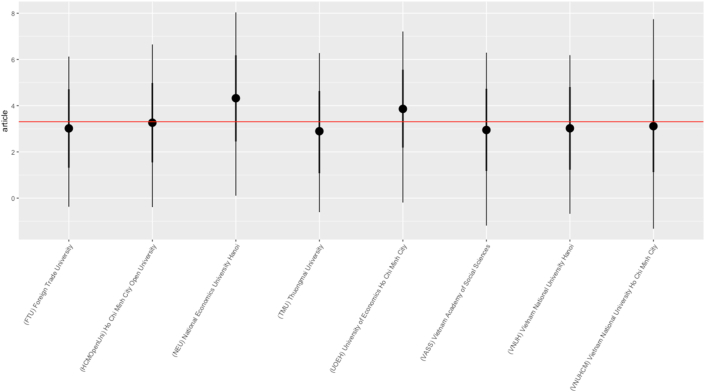
Average number of articles per affiliation.

From Figure 9, we can see that there are two top affiliations above the average line of the total articles, which are NEU and UOEH. The other six affiliations that closely correlate to the average line are FTU, HCMOpenUni, TMU, VASS, VNUH, and VNUHCM.

***RQ6: Is there any relationship between the authors' scientific productivity and their sexes?***

**Figure 10:**
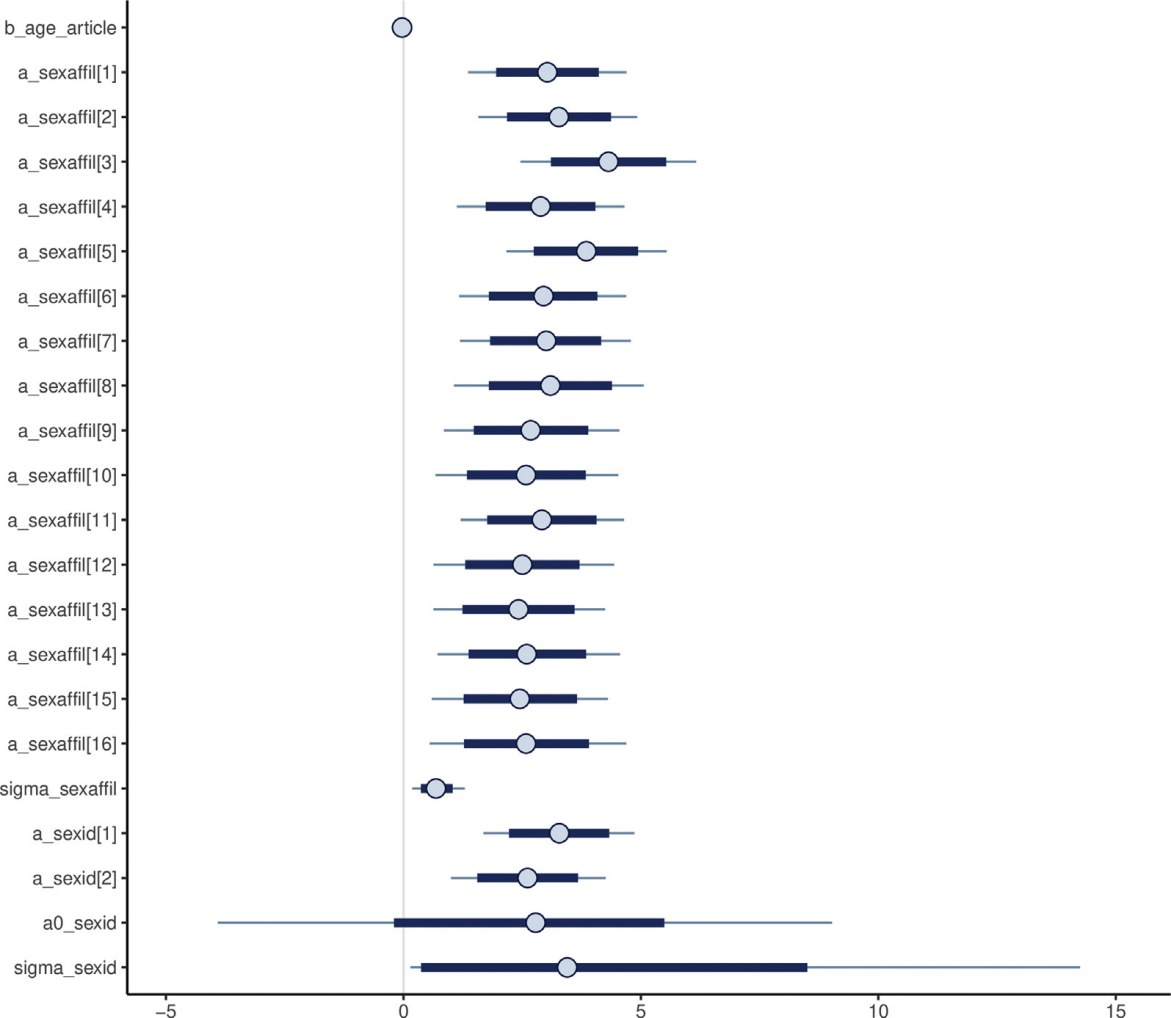
The α_sexaffil_ and α_sexid_ variables in the Article model.

Figure 10 explains the relationship between the number of articles and authors’ sex using the parameters of α_sexaffil[1]_ to α_sexaffil[16]_ and α_sexid[1]_ and α_sexid[2]_. The parameter of α_sexaffil[3]_ is the highest (Mean = 4.32; SD = 0.95), which belongs to male authors and the parameter of α_sexaffil[13]_ is the lowest (Mean = 2.45; SD = 0.94), which belongs to female authors.

**Figure 11:**
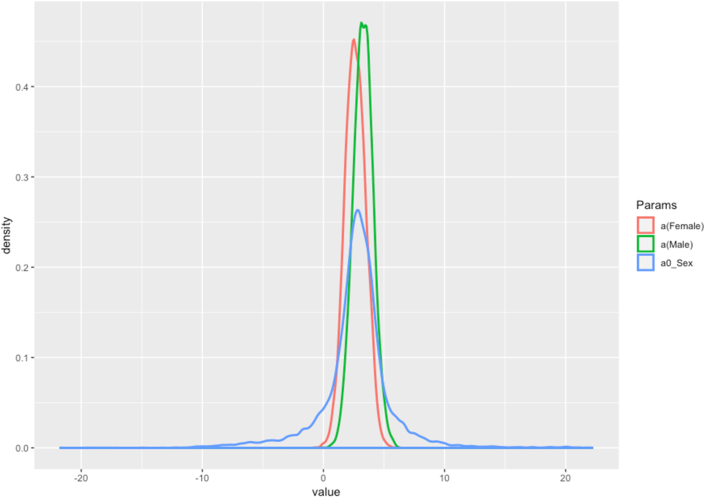
Posterior coefficients of the Article model.

Figure 11 displays the density and value of sex to the authors' scientific productivity. Overall, the male authors (α_sexid[1]_ = 3.28; SD = 0.82) are more likely to publish more than their female counterparts (α_sexid[2]_ = 2.63; SD = 0.85). However, the difference is relatively small. The gap is fairly small. All the parameters lie in the negative zone of Figure 11 value's bar, representing a low probability of association between sex and the authors' scientific productivity. Besides, the distribution of male and female variables is narrow with high density, which indicates a minor association between sex and the authors' scientific productivity with a small variance.

**Figure 12:**
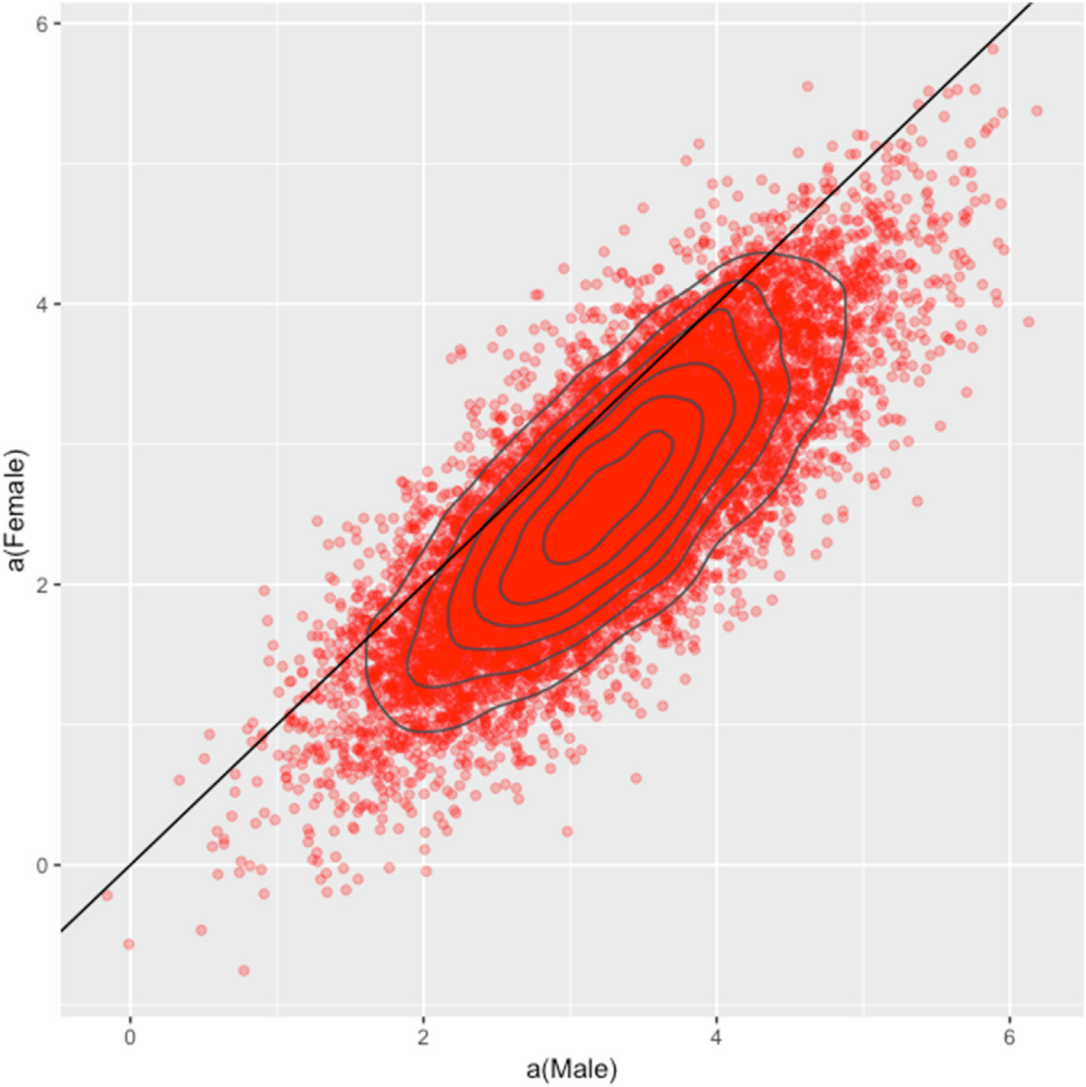
Pairing Female and Male parameters.

Figure 12 shows that both coefficients are positive, which indicates that there is scientific productivity in both male and female authors. At the most concentrated point, the constant coefficient of α_Female_ is smaller than the constant coefficient of α_Male_. Therefore, male authors are more likely to have more publications than female authors.

## 5 Discussion

### 5.1 Top universities and research institutes in Vietnam's economic discipline and their productivity levels

Out of the top 8 affiliations in the economics discipline, only one research institute is found, along with seven universities. The findings that researchers with top universities-affiliated are having greater scientific productivity than institutes-affiliated in Vietnamese economic discipline; with the highest scientific performance recorded from male authors of National Economics University Hanoi (NEU) and the lowest scientific performance from female authors of the University of Economics Ho Chi Minh City (UOEH).

To shed light on the differences in the productivity between universities and research institutions, Vietnam's higher education system needs to be further explained. Before the 1980s, the system consisted of universities and research institutions assigned to conduct separate functions, as influenced by the Soviet model. The former focused primarily on teaching, while the latter's main responsibilities lie in producing scholarly works (Nguyen, 2014). However, after the *Đổi Mới*, i.e., Renovation in 1986, the roles and functions of universities have expanded considerably to include not only teaching but also researching (Vuong et al., 2019). However, it is still stated that research and development activities remain traditionally in research institutions, and most academics in universities are not actively engaged in these activities (World Bank, 2008). As an attempt to improve this situation, the Ministry of Education and Training issued a circular in 2017 which requires all doctoral students to have at least two publications indexed in Scopus and/or Web of Science, and the supervisor also needs to have international publications.

The result of this study ties with previous studies, which find that authors working at universities have much higher scientific output than those affiliated with research institutions (Vuong et al., 2019). This raises concerns about research institutes, whose main function is to conduct research; however, their productivity is questionable. One plausible explanation might be that researchers from Vietnamese universities have more chances to corporate and receive funds from foreign partners, which explains the imbalance in scientific output from universities-based affiliation and institutes-based affiliation. Hence, the government should take into account the need to encourage both affiliated authors to increase their scientific productivity sustainably, such as create more regulations to attract foreign funds for economic research in Vietnam, raising the awareness of economic studies in students, or invest in institutes-based research projects. Also, the need to professionalize the research management system in Vietnam and particularly in universities is paramount so that academics have the opportunities to work in a more organized and better-funded research culture (Nguyen and Meek, 2016). The policymakers should also note the interesting trend that university-affiliated researchers turn out to publish even more than their counterparts at research institutions, even though their primary responsibility lies in teaching. From this finding, strategies to help maximize research capacity from both sides can be proposed.

### 5.2 Age and scientific productivity

Our findings suggest that there might be a decrease in the scientific productivity of older scientists, which is compatible with previous research by Costas et al. (2010). The findings show that 40–44 is the age group with the highest scientific productivity in the significant number of top economic affiliations within the 2008–2019 period. This group will keep playing a crucial role in the scientific development of the Vietnamese economics discipline. As Ph.D. candidates is expected enter the job market in their 30s, then 40–44 years old might have been the greatest period for scientists to boost their scientific productivity because they have been tested adaquately in a highly competitive job market (Donnelly et al., 2019).

Regarding the senior age groups, there are possible reasons for their inactivity in scientific publications. The senior age groups in Vietnam received their training in China or the Soviet Union. Unlike natural science, social sciences and humanities did not translate well between languages and ideologies. Moreover, as social sciences and humanities focus on local problems, the pressure to publish internationally was not as urgent as in other fields. Therefore, seniors’ contributions might appear limited. However, they are crucial in training the next generation and promoting new policies that help push the scientific community forward.

The scientists might need sufficient resources and motivation to keep up their scientific performance for a long time. In summary, the age group is one of the important variables when analyzing scientific productivity.

### 5.3 Sex and scientific productivity

Apart from age, sex is also an important variable in analyzing Vietnamese economic discipline's scientific productivity. The SAGER guideline was used to define the research groups, which are male and female, in this study (Heidari et al., 2016). Our findings show a sex inequality in the scientific productivity of the top eight Vietnamese affiliations. This result is consistent with the previous study of Larivière et al. (2011) that female authors have less productivity in publications and less research age span than male authors. The previous research finds that motherhood, restricted cooperation networks, and access to resources might be the limitation for female authors. In Vietnam, this limitation might also come from the difference in retired age between females and males. According to the World Bank (2009) report, female's retirement age (55 years old) is lower than male's retirement age (60 years old) in Vietnam. Therefore, female authors might have less time to prove their scientific performance at work.

The number of articles from male authors outweighs the number of articles from females in most universities, especially in the University of Economics Ho Chi Minh City (UOEH), National Economics University Hanoi (NEU) – 2 in 8 affiliations with the highest number of published articles. The Government of Vietnam is committed to the Sustainable Development Goals in 2030, which have ‘Gender Equality’ as one of the main goals (United Nation Vietnam, 2018). Therefore, this inequality in scientific performance might need careful consideration from Vietnam authorities to reach the Sustainable Development Goals in 2030.

Results from Bayesian analysis also shed some light on the relationship between sex and Vietnamese economic researchers' scientific productivity. First of all, the findings show that both male and female researchers do have a scientific contribution to their affiliations in the last twelve years. Despite that, our results indicate that males have a higher probability of publications than females; however, the gap is relatively small. It is proven to be only a minor association between sex and the authors’ scientific productivity. In contrast, we can see inequality in the number of articles from the male and female authors, as shown in the descriptive data analysis. The difference in the number of articles is fairly enormous, with male authors outweigh female authors in all the affiliations. Hence, our findings suggest that the number of articles might not be the only element to evaluate the relationship between sex and the scientific productivity of a researcher.

### 5.4 Authorship, collaboration, and scientific productivity

In the 2008–2016 period, there is only a small increase in the number of new authors per year. However, in 2017, there has been an explicit increase of new authors in many affiliations, such as Ho Chi Minh City Open Universit. Circular 08/2017/TT-BGDDT in 2018, which requires Ph.D. candidates to publish at least two articles in ISI/Scopus journals (Vuong et al., 2020d), might have been the motivation for the new authors to publish more articles. Since 2017, the number of lead authors in NEU increases per year, significantly rockets to 21 authors in 2017 with 17 published articles. There is an upward growth of new authors in most affiliations, especially NEU, as the leading affiliation with more than 25 new authors in 2019.

Our findings also indicate co-authorships in all of the top eight affiliations in the Vietnamese economic discipline. There is evidence for the correlation between co-author rate and the scientific productivity of these eight affiliations. Co-authorships might be one of the important factors to increase researchers’ scientific productivities from universities. The universities with high co-author rates have better scientific performance in top affiliations such as NEU, UOEH, or VNUHCM. This result is compatible with previous research by Piro et al. (2013), which suggests the importance of collaborative authorship in scientific productivity. That means the increase in extensive collaboration between authors might continue to be common in the Vietnamese economics discipline.

### 5.5 Journal impact factor and scientific productivity

On the other hand, the results show that the significant number of articles from the top eight affiliations has JIF = 0, only belongs to Scopus or Emerging Sources Citation Index (ESCI). Only UOEH and Thuongmai University have articles with a JIF of 5 among the top list of affiliations. Scientific productivity is based not only on the number of articles but also on producing quality research. Our study suggests that the Vietnamese economic discipline lacks quality research, which can be qualified by international standards, despite having a great number of researches in the last twelve years. From another perspective, regarding the controversial nature of the metric, previous research by Bertuzzi and Drubin (2013) claimed that the IF does not assess the creativity or value of any individual study. The implementation of IF is more in scaling up the subscribership of the publications in which a paper appears or the institutions it keeps. The metric is argued to be designed to indicate the quality of journals, and therefore should not be used as a proxy to assess the quality of any single paper or its authors (Callaway, 2016). Therefore, it is suggested that the policy-making and institutions’ internal regulations should consider using a combination of IF and other metrics to evaluate the quality of the scholarly works.

## 6 Conclusion

Our findings show a correlation between a researcher's affiliation, sex, and their scientific productivity. In the Vietnamese economics discipline, authors from top universities-affiliated have higher scientific output than institutes-affiliated. However, we also find that the number of published articles might not be the only point to evaluate the researchers' scientific productivity. The scientific output might be measured as the relative productivity and the authors' contribution to each article, rather than solely the number of publications. However, as the international standard for publication is becoming more wide-spread and stricter, the researchers might be concerned about the number of publications to prove their scientific performance. To sustain scientific productivity, we suggest not only thinking about the quantity of the papers but also the quality of the research.

The study has several limitations (Vuong, 2020). Firstly, the absence of the multi-affiliated authors' evaluation might be a potential research area in the future. Secondly, the study used JIF as one of the metrics; it should be noted that JIF does not indicate research quality. Our research contributes to the development of the Vietnamese economics discipline and the Social Sciences and Humanities community. The findings call for further studies from Vietnamese researchers to continuously evaluate the complex relationship between the authors’ affiliations and their scientific productivity.

## Declarations

### Author contribution statement

Q. H. Vuong: Conceived and designed the experiments; Performed the experiments; Analyzed and interpreted the data; Contributed reagents, materials, analysis tools or data.

A. T. Bui: Conceived and designed the experiments; Analyzed and interpreted the data; Contributed reagents, materials, analysis tools or data.

M. T. Ho, M-T. Ho: Analyzed and interpreted the data; Contributed reagents, materials, analysis tools or data; Wrote the paper.

T. H. Pham, T. H. Vu: Performed the experiments; Wrote the paper.

H. H. Pham, A. D. Hoang: Analyzed and interpreted the data; Wrote the paper.

V. P. La: Performed the experiments; Analyzed and interpreted the data; Contributed reagents, materials, analysis tools or data.

### Funding statement

This work was supported by the National Foundation for Science and Technology Development (502.01-2018.19).

### Data availability statement

Data associated with this study has been deposited at Open Science Framework: URL: https://osf.io/h3esk/; DOI: https://doi.org/10.17605/OSF.IO/H3ESK.

### Declaration of interests statement

The authors declare no conflict of interest.

### Additional information

No additional information is available for this paper.

## Appendix A Supplementary data

Supplementary.docx Supplementary.docx
