# Supplementary material for: Top economics universities and research institutions in Vietnam: evidence from the SSHPA dataset
Source: Heliyon. 2021 Feb 17;7(2):e06273. doi: 10.1016/j.heliyon.2021.e06273 (PMC7903316; doi:10.1016/j.heliyon.2021.e06273)
Supplement: Supplementary.docx [file mmc1.docx]

**Supplementary for**

**Top economics universities and research institutions in Vietnam: Evidence from the SSHPA dataset**

**SexAffil variables:**

| data1$affilid <- as.numeric(data1$affil)  data1$sexaffil <- factor(paste0(data1$sexid,”_”,data1$affilid)) |
| --- |

**Examples of R code:**

| **# Design the model**  model <- bayesvl()  model <- bvl_addNode(model, “article”, “norm”)  model <- bvl_addNode(model, “sexaffil”, “cat”)  model <- bvl_addNode(model, “sexid”, “cat”)  model <- bvl_addNode(model, “age”, “norm”)  model <- bvl_addArc(model, “age”, “article”, “slope”)  model <- bvl_addArc(model, “sexaffil”, “article”, “varint”)  model <- bvl_addArc(model, “sexid”, “sexaffil”, “varint”) |
| --- |
